# Abscisic acid mediated strawberry receptacle ripening involves the interplay of multiple phytohormone signaling networks

**DOI:** 10.3389/fpls.2023.1117156

**Published:** 2023-01-30

**Authors:** Bai-Jun Li, Yan-Na Shi, Hao-Ran Jia, Xiao-Fang Yang, Yun-Fan Sun, Jiao Lu, James J. Giovannoni, Gui-Hua Jiang, Jocelyn K. C. Rose, Kun-Song Chen

**Affiliations:** ^1^ College of Agriculture and Biotechnology, Zhejiang University, Zijingang Campus, Hangzhou, China; ^2^ Zhejiang Provincial Key Laboratory of Horticultural Plant Integrative Biology, Zhejiang University, Zijingang Campus, Hangzhou, China; ^3^ State Agriculture Ministry Laboratory of Horticultural Plant Growth, Development and Quality Improvement, Zhejiang University, Hangzhou, China; ^4^ Institute of Horticulture, Zhejiang Academy of Agricultural Sciences, Hangzhou, China; ^5^ Plant Biology Section, School of Integrative Plant Science, Cornell University, Ithaca, NY, United States; ^6^ United States Department of Agriculture – Agricultural Research Service and Boyce Thompson Institute for Plant Research, Cornell University, Ithaca, NY, United States

**Keywords:** strawberry, phytohormone signalings, abscisic acid, coexpression network, fruit qualities, ripening

## Abstract

As a canonical non-climacteric fruit, strawberry (*Fragaria* spp.) ripening is mainly mediated by abscisic acid (ABA), which involves multiple other phytohormone signalings. Many details of these complex associations are not well understood. We present an coexpression network, involving ABA and other phytohormone signalings, based on weighted gene coexpression network analysis of spatiotemporally resolved transcriptome data and phenotypic changes of strawberry receptacles during development and following various treatments. This coexpression network consists of 18,998 transcripts and includes transcripts related to phytohormone signaling pathways, MADS and NAC family transcription factors and biosynthetic pathways associated with fruit quality. Members of eight phytohormone signaling pathways are predicted to participate in ripening and fruit quality attributes mediated by ABA, of which 43 transcripts were screened to consist of the hub phytohormone signalings. In addition to using several genes reported from previous studies to verify the reliability and accuracy of this network, we explored the role of two hub signalings, small auxin up-regulated RNA 1 and 2 in receptacle ripening mediated by ABA, which are also predicted to contribute to fruit quality. These results and publicly accessible datasets provide a valuable resource to elucidate ripening and quality formation mediated by ABA and involves multiple other phytohormone signalings in strawberry receptacle and serve as a model for other non-climacteric fruits.

## Introduction

Fleshy fruits can be classified into those that exhibit either climacteric or non-climacteric ripening: the former type involves a peak of respiration and emission of the gaseous hormone ethylene, which acts as the main regulator of this process, while the latter does not. The phytohormone abscisic acid (ABA) can play either a dominant or supportive role in modulating non-climacteric and climacteric fruit ripening, respectively (Kou et al., 2021a). Although many studies have reported that the ABA controls non-climacteric fruit ripening and influence fruit quality traits (Kou et al., 2021b; Li et al., 2022a), there is limited understanding of this process compared with that of ethylene in climacteric fruit (Wang et al., 2022a). Better elucidation of mechanisms of ABA-mediated fruit ripening has considerable potential for enhancing our understanding of both climacteric and non-climacteric ripening and for developing novel traits and varieties, especially concerning non-climacteric fruit.

Strawberry (*Fragaria* spp.) fruit is a pseudocarp that consists of a receptacle with many achenes (true fruit) embedded in the epidermis. It has typical characteristics of non-climacteric fruit and modern cultivated strawberry (*Fragaria* × *ananasssa*) represents a particularly important fruit crop (FAO, 2020). Strawberry has also been adopted as an experimental model for non-climacteric fruit, which is reflected in the development of effective transgenic systems and ever-growing genomic resources (Edger et al., 2019; Zhou et al., 2020; Kou et al., 2021b; Liu et al., 2021). Through the development of such resources, genes that affect strawberry fruit quality, including coloration (i.e. anthocyanin biosynthesis) (Fischer et al., 2014; Castillejo et al., 2020; Gao et al., 2020), sugar accumulation (Jia et al., 2013a; Jia et al., 2016), aroma (Raab et al., 2006; Medina-Puche et al., 2015; Molina-Hidalgo et al., 2017) production and softening (Molina-Hidalgo et al., 2013; Paniagua et al., 2016), have been identified. Moreover, members of transcription factor (TF) families have been revealed as inducers or suppressors of strawberry fruit ripening (Li et al., 2022a), including the MADS genes *SHATTERPROOF*-like (*FaSHP*; Daminato et al., 2013), *FaMADS1a* (Lu et al., 2018), *FaMADS9* (Vallarino et al., 2020), and *FveSEP3* (Pi et al., 2021), and the *NAC*, *Ripening Inducing Factor* (*FaRIF*; Martín-Pizarro et al., 2021). The expression levels of most of these genes are affected by ABA, and the ABA biosynthetic pathway in strawberry fruit has been also well described (Li et al., 2022a).

Notably, most studies investigating the roles of phytohormones in fruit development have used exogenous hormone treatments. In strawberry, auxin production, which supports the development of the receptacle, and which is antagonistic to ABA (Li et al., 2022a), occurs in the achenes (Thompson, 1969). Accordingly, removing achenes from the receptacle causes reduced auxin levels and, consequently, an elevation in ABA levels and a promotion of receptacle ripening in the late developmental stage (Li et al., 2022b). This experimental manipulation therefore provides a means to study ABA-associated receptacle ripening, in addition to the use of exogenous ABA treatments. In summary, strawberry provides an excellent model system in which to characterize the ABA-mediated fruit ripening of non-climacteric fruit, analogous to the adoption of tomato (*Solanum lycopersicum*.), as principal model for ethylene-regulated climacteric ripening (Liu et al., 2020; Fenn & Giovannoni, 2021; Kou et al., 2021b).

Multiple phytohormone signaling genes participating in strawberry ripening regulated by ABA have been documented (Gu et al., 2019; Fenn & Giovannoni, 2021; Wang et al., 2022a), and ABA can act synergistically or antagonistically with auxin, gibberellins (GAs), ethylene, and jasmonic acids (JAs) in strawberry (Li et al., 2022a). In addition, the roles of ABA signaling genes in ripening, including *FaPYR1* (*Pyrabactin resistance 1*; Chai et al., 2011), *FaABI1* (*ABSCISIC ACID-INSENSITIVE 1* encoding a PP2C protein; Jia et al., 2013b), *FaSnRK2.6* (*SNF1-related protein kinase 2.6*; Han et al., 2015), and *FaABAR* (*Magnesium-protoporphyrin IX chelatase H subunit*; Jia et al., 2011), have been well characterized. These results are consistent with the existence of complicated strawberry ripening mechanisms, involving multiple phytohormones signalings, and disproportionately influenced by a predominant ABA-signaling pathway. However, there are much remains to be learnt about the hub phytohormone signalings in strawberry and additional non-climacteric fruit mediated by ABA.

In this study, we investigated phytohormone signaling pathways in the strawberry receptacle ripening mediated by ABA, using transcriptome profiling of the receptacle at three developmental stages from unripe to ripe and following changes of ABA levels *via* exogenous and removing achenes treatments. Following weighted gene coexpression network analysis (WGCNA), we described a coexpression network and predicted the hub phytohormone signaling genes in ABA-mediated receptacle ripening. Additionally, we identified two hub signaling genes, *small auxin-up RNA*s (*FaSAUR1* and *FaSAUR2*), shown by transient RNA interference (RNAi), to promote receptacle quality formation. Finally, the full-length transcript sequences and their spatiotemporally resolved expressional profiles provide new insights into ABA mediating associated phytohormone signalings in ripening strawberry fruit.

## Materials and methods

### Plant materials and sampling

*Fragaria* × *ananassa* ‘Yuexin’ fruit were sampled at the green (G, green receptacle embedded green achenes), turning (T, pale green receptacle embedded with some brown or green achenes) and half red (HR, half a receptacle with some red and brown achenes) stages (**Supplementary Figure S1**), achenes were removed as described below from a subset of fruit, and then the samples were immediately frozen in liquid nitrogen and stored at –80°C. All ‘Yuexin’ fruit used in this study were grown in a Zhejiang Academy of Agricultural Sciences plastic greenhouse (Zhejiang, China) under natural light with daytime and night-time temperatures of 10-24°C.

### Removal of achenes and exogenous hormone treatments

The fruit were carefully removed half achenes using a tweezer along the centra axis from the receptacles at G stage. Water (sterile ultrapure water as control for exogenous hormone treatments) or NAA (500 μM; Sigma-Aldrich, USA), and ABA (500 μM; Sigma-Aldrich, USA) was then injected into the whole receptacles, using a 1 mm injection syringe. NAA and ABA were dissolved in sterile ultrapure water to a final concentration of 500 μM. Each of exogenous hormone or water treatment had three biological replicates.

### Determination of ABA content

Each freeze-dried sample (0.1-0.2 mg) was placed in a 2 ml centrifuge tube, 1 ml of acetonitrile (Sigma-Aldrich, USA) containing 0.1% formic acid was added and the sample was incubated for12 h at 4°C. The centrifuge tube was then ultrasonicated in an ice water bath for 10 min and centrifuged at 13,500 g and 4°C for 10 min. The supernatant was then collected and filtered through a 0.22 μm membrane filter (organic phase) into a 1.5 ml centrifuge tube. The supernatant was dried in a stream of nitrogen gas. The dried sample was then redissolved in 50 μl acetonitrile, ultrasonicated in an ice water for 5 min and centrifuged at 13,500 g and 4°C for 5 min, then 40 μl of the supernatant was transferred to a brown chromatography vial sample bottle and subjected to high performance liquid chromatography (HPLC, Waters e2695 Separations Module, Waters, USA). A SunFire C18 5μm, 4.6 × 250 mm, column (Waters, USA) was selected and the following program use: sample introduction for 10 μl, sample temperature at 8°C, and maximum and minimum psi of 4,000 and 0, respectively. The mobile phase consisted of 0.1% formic acid (Phase A) and acetonitrile containing 0.1% formic acid (Phase B) and the program was A: B (95: 5) at 0 min; A: B (80: 20) at 20 min; A: B (35: 65) at 40 min; A: B (0: 100) at 40.5; A: B (0: 100) at 45.5 min; A: B (5: 95) at 46.5; A: B (5: 95) at 52 min. An ABA standard (Sigma-Aldrich, USA) was used for identification and quantification.

### Measurement of qualities related to receptacle ripening

The furanone of the receptacle was extracted and measured using a gas chromatography mass spectrometry (GC/MS, Agilent 7890A GC System, Agilent Technologies Inc., MA, USA) as previously described (Zhang et al., 2018). Sugars were extracted and estimated as in our previously study (Li et al., 2022c) and anthocyanins were extracted and measured using an ultraviolet spectrophotometer (UV-2600, SHIMADZU, Japan) as previously described (Wang et al., 2022b).

### Total RNA extraction and quality assessment

All freeze-dried samples were powdered in liquid nitrogen and 50 mg samples used to extract total RNA using a CTAB method (Shan et al., 2008). The purity and concentration of RNA samples were measured using a NanoDrop™ One/OneC system (Thermo Fisher Scientific, MA, USA), and the integrity of each RNA samples were estimated using an Agilent 2100 Bioanalyzer (Agilent Technologies Inc., CA, USA) and agarose gel electrophoresis.

### PacBio *Iso*-*Seq* library preparation, sequencing and data analysis

To obtain full-length transcriptome sequences expressed during receptacle development, the total RNA of the basal and apical of the receptacle from G, T, HR stages were fully mixed in equal quantity to construct PacBio *Iso*-*Seq* libraries, and sequenced using a PacBio Sequel2 platform. The library preparation and sequencing were performed as previously described (Li et al., 2020).

The SMRT Link v8.0.0 pipeline (Gordon et al., 2015) was used to generate unique full-length transcriptome sequences (isoforms) from the raw sequence data. Briefly, the circular consensus sequence (CCS) reads were first extracted from the BAM file and the full-length (FL) reads (i.e., the CCS reads containing 5’ primer, 3’primer and poly A structures) were then obtained from CCS reads. Second, the primers, barcodes, poly A tail trimming and concatemer of full passes were removed from the FL reads to obtain full-length non-chimeric (FLNC) reads. Subsequently, the FLNC reads were clustered hierarchically using Minimap2 (Li, 2021) to generate the consensus FLNC reads. Third, the Quiver algorithm (Gordon et al., 2015) was used for further correcting the consensus FLNC reads to obtain the high-quality consensus FLNC reads. Finally, CD-HIT-v4.6.7 (Li & Godzik, 2006) with a threshold of 0.99 identity was used to eliminate redundancy from high-quality consensus FLNC reads to obtain isoforms.

The isoforms were annotated by BLAST searches of the nonredundant protein (NR) database (http://www.ncbi.nlm.nih.gov), the Swiss-Prot protein database (http://www.expasy.ch/sprot), the Kyoto Encyclopedia of Genes and Genomes (KEGG) database (http://www.genome.jp/kegg), COG/KOG database (http://www.ncbi.nlm.nih.gov/COG) with an E-value threshold of 1e−5. We used ANGEL (Shimizu et al., 2006) to predict the coding sequences (CDSs), protein sequences, and UTR sequences of the isoforms.

### Illumina transcriptome (*RNA*-*Seq*) library preparation, sequencing and expression level estimation

The total RNA of the basal and apical of the receptacle from G, T, HR stages, and the achened and de-achened sides of the receptacles with the treatments after 9 and 12 days were used to generate RNA-Seq data using an Illumina HiSeq™ 4000, The RNA-Seq library construction, sequencing, and the clean data acquisition from the raw data were performed as previously described (Li et al., 2020). The clean data from each sample were mapped into the isoform set to estimate expression levels *via* FPKM of isoforms using RSEM (Li & Dewey, 2011).

### Weighted gene coexpression network analysis

The WGCNA was constructed using WGCNA (v1.47) package in R (Langfelder & Horvath, 2008). The expression values of isoforms (FPKM ≥ 7) were used to establish weighted gene coexpression modules under the automatic network construction function blockwiseModules with default settings, and the power was 5; the TOMType was unsigned; the mergeCutHeigh was 0.9; the isoform number of minModuleSize was 50. Finally, the isoforms were clustered into 21 modules. The correlation between modules and traits were estimated using the Pearson’s correlation analysis (http://omicshare.com/tools/) between values of module eigengene and phenotypic data, which was displayed using a heatmap analysis. Additionally, the Pearson’s correlation analysis between GS (Gene significance value, a Pearson’s correlation between expression level of each gene in a module and phenotypic data) and MM (Module membership, a Pearson’s correlation between expression level of each gene in a module and the values of module eigengene) was used to further identify the most relevant modules associated with receptacle ripening. The coexpression network of each module was visualized using Cytoscape v3.3.0 (Shannon et al., 2003).

### Phylogenetic and heatmap analyses

To identify the phylogenetic relationships between FaSAUR and SAUR proteins from other plants, a Neighbor-joining tree was constructed with bootstrap values evaluated from 1,000 replicate runs using MEGA7 (Kumar et al., 2016). The alignment of the amino acid sequences was performed using Clustal W (Larkin et al., 2007). All heatmap analyses in this study were conducted using Omiscshare tools (http://omicshare.com/tools/).

### Transient silencing of *FaSAUR*s by *Agrobacterium* infiltration

To verify the function of *FaSAUR1* and *FaSAUR2*, we used RNA interference (RNAi) to silence these two genes in ‘Yuexin’ receptacles, using the RNAi methodology as previously described (Shi et al., 2021) with some modifications. Briefly, the partial fragments of *FaSAUR1* and *FaSAUR2* (**Supplementary Figure S5**) were ligated into pHELLSGATE 2 vector (Shi et al., 2021) using BP Clonase (Invitrogen, MA, USA) to construct *35Spro : FaSAUR1*-RNAi and *35Spro : FaSAUR2*-RNAi, respectively. The recombinant plasmids were transformed into *Agrobacterium tumefaciens* GV3101 by electroporation^46^. After incubation, the GV3101 suspension containing the RNAi vector solution were centrifuged and the cell pellets were resuspended in liquid infection medium (sterile ultrapure water containing 10 mM 2-morpholinoethanesulphonic acid, 10 mM MgCl_2_, and 150 μM acetosyringone) to an OD_600_ = 1 (Zhang et al., 2020). The final suspensions were injected into the whole receptacle at the T stage using a syringe. The infected fruits were then cultivated in the greenhouse, photographed and sampled. The primers of RNAi fragments of *FaSAUR1* and *FaSAUR2* were designed based on transcriptome data (**Supplementary Table S1**).

### Statistical analysis

Statistical significance was assessed with Student’s paired *t*-test using Omiscshare tools (http://omicshare.com/tools/).

## Results

### Evaluation of ABA levels and quality metrics in the receptacle during development and following removing achenes with exogenous ABA treatments

Using *F*. × *ananasssa* ‘Yuexin’ as a model allo-octoploid strawberry cultivar, receptacle ripening progressed from the apical to the basal region based on progression of color change (**Supplementary Figure S1**). Levels of ABA and additional ripening-related compounds increased in the whole receptacle during development, and were notably higher in the apical as compared to the basal region of the fruit (**Supplementary Figure S1**). This was especially clear for ABA at the T and HR stages, while there was a similar difference in the other three ripening-related compounds, including anthocyanin, HDMF, and sugars, only at HR, consistent with ABA acting as a ripening promoter of receptacle tissue (**Supplementary Figure S1**).

**Figure 1:**
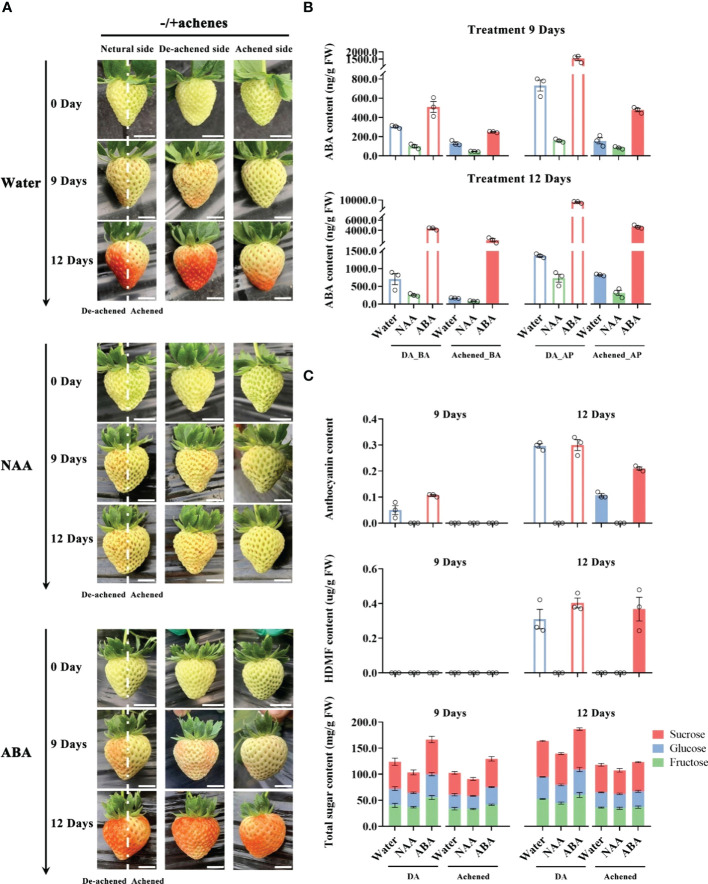
Levels of ABA, HDMF, sugars, and anthocyanins the receptacle after removing and exogenous hormone treatments. **(A)** Whole receptacle from which half the achenes were removed along the central axis at the G stage were injected with water, ABA (500 μM), or naphthylacetic acid (NAA, 500 μM), and photographed after 0, 9 and 12 days. Photographs are shown of individual fruit from three perspectives: ‘neutral side’, with the achenes removed from the left side of the dotted lines (‘De-achened side’), or from the perspectives of the left or right sides of the dotted lines (‘Achened side’), as indicated. Scale bars = 1 cm. **(B)** ABA levels in the different parts of the receptacles at 6 and 9 days after the treatments. The DA, Achened, BA, and AP in labels represent the de-achened and achened sides, basal and apical parts of receptacles, respectively. **(C)** Levels of ripening-related compounds in the DA and achened sides of the receptacles at 6 and 9 days after the exogenous hormone treatments. DA and Achened indicate the de-achened and achened sides of the receptacles, respectively. The data values are the mean ± SD of three biological replicates.

To confirm and deeply explore ABA as a dominant role in strawberry receptacle ripening, we removed half of the achenes from the receptacle at the G stage and injected water, ABA, or the synthetic auxin naphthylacetic acid (NAA) due to auxin as a repressor for ABA biosynthesis^24^, into the receptacle. After injecting water or ABA, the pigmentation on the side of receptacle from which the achenes had been removed (‘de-achened side’) developed more rapidly than the side with achenes (‘achened side’), while there was no visible difference between the two sides following the NAA treatment (**Figure 1A**), consistent with achene-derived auxin inhibiting ripening. On day 9 after treatment, the de-achened side of receptacle was more pigmented after the ABA treatment than after the water treatment, and at day 12 the achened side was fully red after ABA treated fruit, but only half-red after the water treatment (**Figure 1A**). These results support ABA as a ripening promoter of receptacle tissue.

The receptacles of the treated fruit were divided into four parts, corresponding to the basal and apical parts of de-achened (DA_BA and DA_AP) and achened sides (Achened_BA and Achened_AP), and ABA levels were measured. ABA contents in the de-achened side, including basal and apical parts, were higher than those in the achened side after treatments, which suggested that removing the achenes promoted ABA biosynthesis (**Figure 1B**). Moreover, ABA levels in the basal parts of the de-achened and achened sides were lower than those in the apical parts. The receptacle with NAA treatment had the lowest ABA contents among the various treatments and fruit regions, again suggesting that auxin produced by the achenes acts as a repressor of ABA, and consistent with the non-coloration phenotype (**Figure 1A**).

The levels of ripening-related compounds in the de-achened and achened sides were quantified to investigate the degree of ripeness. Anthocyanin accumulated in the de-achened side of the receptacle following water and ABA treatments at day 9, and ABA treatment resulted in the highest levels (**Figure 1C**). At day 12, anthocyanin levels were similar in the de-achened tissues under ABA and water treatments, while in the achened side they were higher under ABA treatment than with water. The receptacle following NAA treatment showed no evidence of anthocyanin accumulation, consistent with the lack of coloration (**Figure 1A**). The important strawberry aroma compound, HDMF, was detected in both de-achened and achened tissues of the receptacles under ABA treatment and in the de-achened side under water treatment at 12 days (**Figure 1C**). In addition, the total sugar content of the de-achened sides were higher than in the achened sides, and samples from the ABA and NAA treatments had the highest and lowest contents, respectively, among the different treatments (**Figure 1C**). Together, these results demonstrate a positive correlation between the measured ripening-related compounds (**Figure 1C**) and ABA accumulation (**Figure 1B**).

### Transcriptome sequencing and analyses of the receptacles during development and following hormone treatments

The genome of strawberry (*F*. × *ananassa* ‘Camarosa’) comprises four parental subgenomes (Edger et al., 2019), which complicates calculating expression levels and profiling gene expression using RNA-Seq. To obtain the full-length transcript sequences (Isoforms) expressed during receptacle development, the total mRNAs extracted from basal and apical parts of receptacles at the G, T, HR stages were pooled and sequenced using the PacBio platform (**Supplementary Figure S2**). This resulted in the identification of 52,455 transcript isoforms. The different parts of the receptacles during development and under the treatments (a total of 54 samples), were subjected to RNA-Seq using an Illumina platform, and corresponding gene expression profiles were generated (**Supplementary Figure S2**). Approximately 375.50 gigabytes (GBs) of cleaned sequence data were produced and mapped to the isoform set, with a high mapping ratio (97.2–97.5%). Thus, almost all genes expressing during receptacle development were identified. The gene expressional profiles consisted of three categories: spatial expression, temporal expression during development, or related to changes in ABA levels manipulated by removing achene and exogenous treatments, and these variable and complicated expression patterns can deeply explore potential relationship between phenotypes and gene expressions, and between gene expressions. And then, the isoforms were clustered into 21 modules according to their fragments per kilobase per million (FPKM) using WGCNA (**Supplementary Figure S2**). Among these modules, the turquoise module had the highest number of isoforms (7,246), while the lowest number was in the grey module (4) (**Supplementary Figure S2**).

**Figure 2:**
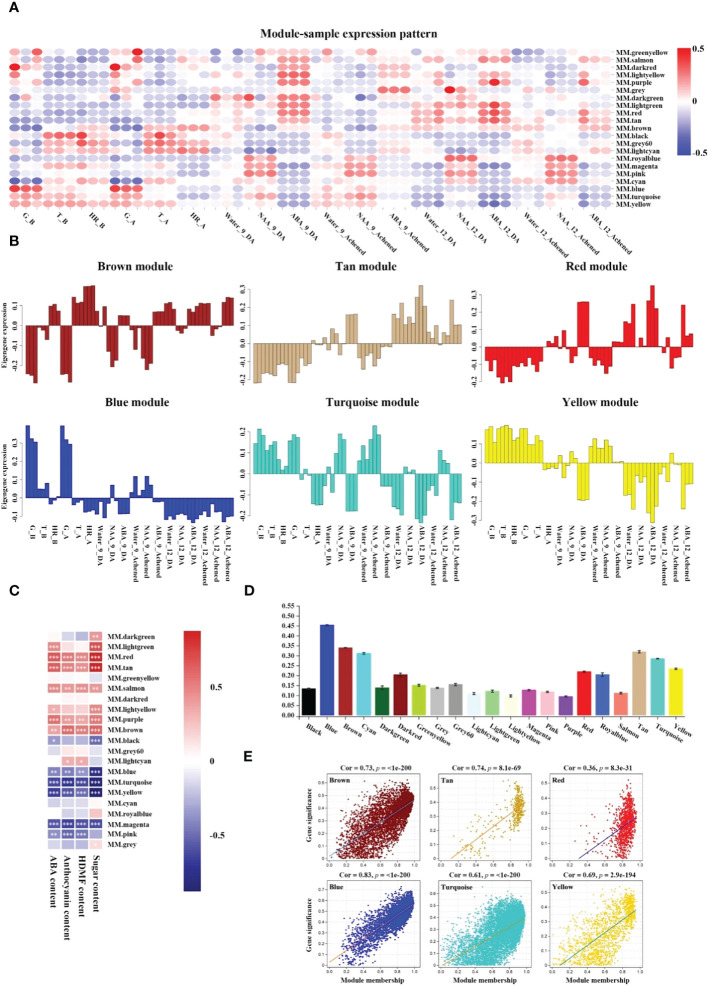
Screening positive and negative modules relevant to receptacle ripening. **(A)** Heatmap analysis of module expression pattern in samples based on gene expression profiles. The red and blue colors denote up- and down-regulation of gene expressions in the samples, respectively. **(B)** Histograms of expression patterns of six modules in each sample. **(C)** The Pearson correlation between expression profiles of genes in modules and ABA, anthocyanin, HDMF, and sugar contents, respectively. According to Student’s paired t-test, white ‘*’, ‘**’, and ‘***’ in the heatmap represent P < 0.05, P < 0.01, and P < 0.001, respectively. **(D)** The mean of the Pearson correlation between the expression profiles of genes in each module and physiological indices. **(E)** Analysis of correlation between gene significance, the correlation between each isoform and ABA level, and module membership, the correlation between expressional profile of each isoform and module. *P* values were analyzed using a Student’s paired *t*-test.

The above data sets were used to identify the modules that had a high correlation with receptacle ripening and these were used to describe the hub phytohormone signaling network regulated by ABA. The expression patterns of the brown, tan, and red modules had a positive relationship with the changes in ABA accumulation, ripening-related compounds qualities, and phenotypes, while the blue, turquoise, and yellow modules were opposite to them (**Figures 1**, **2A, B**; **Supplementary Figure S1**). Moreover, a Pearson correlation analysis further verified that the changes in the physiological indices displayed a significantly positive relationship with the expressional profiles of brown, red, and tan modules and negative with blue, turquoise, and yellow (**Figure 2C**). Subsequently, the Pearson correlations between the expression profiles of genes in each module and physiological indices indicated that these six modules ranked in the top seven of all of the modules (**Figure 2D**). In addition, an analysis of the correlation between gene significance (i.e. the correlation between each isoform and ABA level), and module membership (i.e. the correlation between expressional profile of each isoform and module) showed that the brown, tan, blue, turquoise, and yellow modules had correlation coefficient values > 0.6, with a significance that was higher than others. This suggested that the expression of genes in these modules had a strong relationship with the changes of ABA levels (**Figure 2E**; **Supplementary Figure S3**). Together, these results indicated that the brown and tan modules were positively related to ABA-mediated receptacle ripening, while the blue turquoise, and yellow modules had a highly negative relationship, which suggested that the genes in these modules might participate in receptacle ripening.

### Construction of coexpression networks of phytohormone signaling during receptacle ripening mediated by ABA

**Figure 3:**
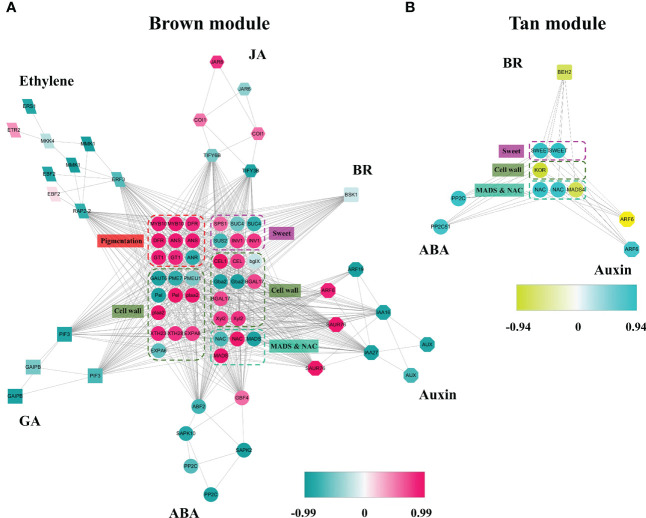
Coexpression networks of ABA and other phytohormone signaling pathways during receptacle ripening, in brown and red modules. **(A)** The coexpression network in the red module is positively related to ripening based on WGCNA. Each of shaped block present an isoform, and the colors denote a positively or negatively relationship with the module as indicated by the scale bar. If more than two isoforms of a gene were detected in the module, only the isoforms with highest and lowest levels of correlation with the module are shown. In the figure, the isoforms associated with phytohormone signaling revolve around the isoforms involved in quality traits, such as pigmentation, softening, and sweetness, as well as TFs including *MADS* and *NAC* genes related to ripening. **(B)** The coexpression network in the tan module positively related to ripening. JA, jasmonic acid; BR, brassinosteroid; GA, gibberellins. The full names of the abbreviations of the isoforms are shown in **Supplementary Dataset S1**.

Based on above results, the red and tan modules had the most positive relationship with receptacle ripening mediated by ABA, while the blue, turquoise, and yellow modules had a negative relationship. The isoforms involved in phytohormone signaling, ripening, and data related to levels of fruit quality related compounds were used to construct coexpression networks related to phytohormones that collectively regulate receptacle ripening. The brown module contained genes associated with six phytohormone: ABA, ethylene, GA, JA, auxin, and brassinosteroids (BR). These genes showed a positive relationship with pigmentation, cell wall metabolism, and sugar accumulation, suggesting that these phytohormone signaling networks may underly the expression of genes that affect commercially important fruit quality traits (**Figure 3A**). We identified sets of ABA (6), auxin (13), ethylene (3), and JA (6) related genes that putatively promote ripening and quality, and equivalent sets plus GA and BR (13, 16, 13, 3, 16, 1 respectively) that putatively suppress ripening and associated traits (**Figure 3A**; **Table 1**). Genes in the anthocyanin biosynthesis pathway and the associated TF regulator, MYB10, have been well studied in strawberry fruit, and their expression is upregulated by ABA (Kadomura-Ishikawa et al., 2015). We detected the expression of several anthocyanin biosynthetic genes and multiple MYB10 genes in the brown module and their expression were up-regulated by ABA, indicating a relationship between the module and ripening and providing validation of the reliability and accuracy of the coexpression network (**Figure 3A**; **Supplementary Figure S4**; **Supplementary Dataset S1**).

**Table 1:** The list of isoforms associated with phytohormone signaling in five modules.

Module	Phytohormones	Positive	Negative	Total members
Brown	ABA	6	13	19
	Auxin	13	16	29
	Ethylene	3	13	16
	JA	6	3	9
	GA	NA	16	16
	BR	NA	1	1
Tan	ABA	3	NA	3
	Auxin	2	NA	2
	BR	NA	1	1
Blue	ABA	NA	11	11
	Auxin	1	23	24
	Ethylene	3	8	11
	JA	1	3	4
	GA	NA	4	4
	BR	NA	13	13
	CTK	NA	1	1
	SA	2	1	3
Turquoise	ABA	4	20	24
	Auxin	8	19	27
	Ethylene	2	32	34
	JA	8	5	13
	GA	NA	5	5
	BR	NA	11	11
	CTK	3	2	5
	SA	NA	1	1
Yellow	ABA	2	1	3
	Auxin	1	2	3
	Ethylene	2	12	14
	JA	1	4	5
	BR	NA	7	7
	SA	2	NA	2

Positive and Negative represent expression profiles of isoforms positively and negatively related, respectively, to receptacle ripening mediated by ABA. NA, not available.

We also determined that the expression of genes, including *sucrose-phosphate synthase* (*SPS*) and *beta-fructofuranosidase/invertase* (*INV*), involved in sugar accumulation, was positively related to the brown module and changes in sugar levels in the receptacle (**Figures 1C**, **3A**; **Supplementary Figure S1**). Additionally, we identified TF genes in the *MADS* and *NAC* families that have been widely associated with ripening (Kou et al., 2021a; Kou et al., 2021b). Specifically, we observed that the expression of two *NAC* genes and one *MADS* gene had a negative relationship with the brown module, while 9 *NAC* genes and 8 *MADS* genes showed a positive relationship (**Figure 3A**). Among these MADS genes, Isoform0048909 has a positive relationship with the brown module and are down-regulated by ABA (**Supplementary Figure S4**). Notably, its predicted amino acid sequence corresponds to FaSHP, which participates in receptacle ripening mediated by ABA (Daminato et al., 2013) (**Supplementary Datasets S1**, **Dataset S2**). In the tan module, which corresponded to a positive correlation with ripening regulated by ABA, one BR signaling gene and three ABA and two auxin signaling genes were negatively and positively correlated with ripening, respectively. These genes were coexpressed with several *NAC* genes and a *MADS* gene possibly participating in the metabolism of sugars and cell walls (**Figure 3B**).

**Figure 4:**
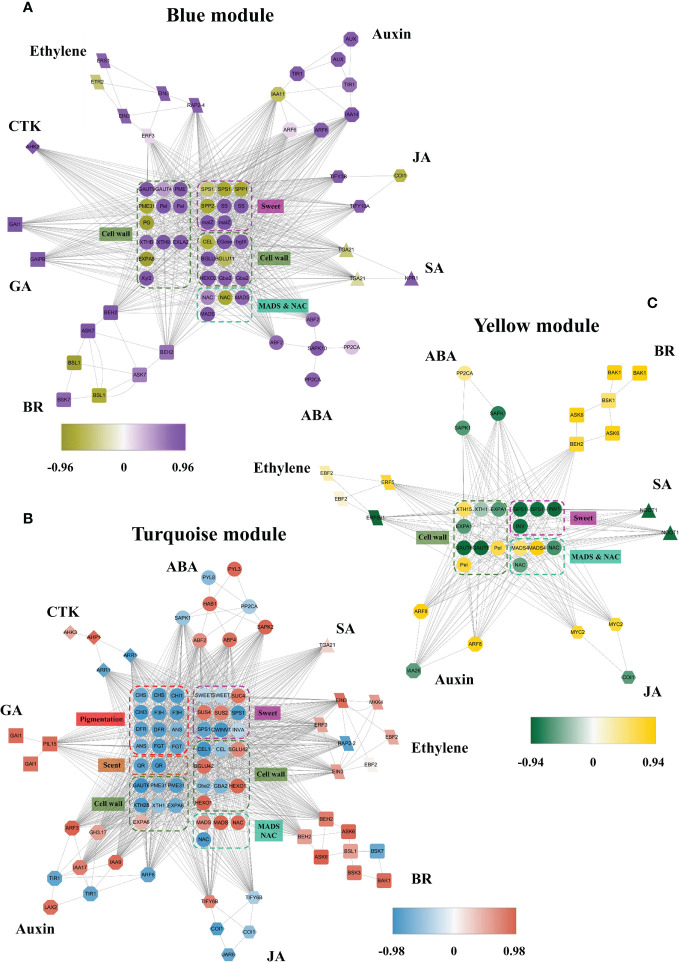
The coexpression networks of ABA and other phytohormone signaling pathways during receptacle ripening in blue, turquoise and tan modules. **(A)** The coexpression network in the blue module was negatively related to ripening based on WGCNA. **(B)** The coexpression network in the turquoise module was negatively related to ripening. CTK, cytokinin; SA, salicylic acid. **(C)** The coexpression network in the yellow module was negatively related to ripening. The full names of the isoforms are shown in **Supplementary Dataset S1**.

Members of phytohormone signaling pathways were also coexpressed in other modules, including blue, turquoise, and yellow, negatively related to receptacle ripening mediated by ABA (**Figure 4**). The coexpression network showed that 40 and 185 members from eight phytohormone signaling pathways were positively and negatively correlated with ripening, respectively (**Table 1**). Among the phytohormones, only GA signaling did not show a positive relationship with receptacle ripening (**Figure 4**; **Table 1**). We also identified 22 *NAC* genes that were positively correlated with ripening, in addition to 3 *NAC* genes and 16 *MADS* genes with a negative relationship. Among these NACs, the predicted amino acid sequences of both Isoform0048861 and 0046736, which belong to the blue module, had ~98% identity to FaRIF, which has been shown to promote for strawberry ripening and is positively regulated by ABA (Martín-Pizarro et al., 2021). This is consistent with the expressional profiles of these two isoforms and the positive relationship between the them and receptacle ripening (**Supplemetary Figure S4**; **Supplementary Datasets S1**, **Dataset S2**). Additionally, the homolog of FaMADS1a, (Isoform0046787), which represses receptacle ripening and is negatively regulated by ABA at the transcriptional level (Lu et al., 2018), was present in the turquoise module and its expression was repressed by ABA (**Supplementary Figure S4**; **Supplementary Dataset S2**).

We also identified genes involved in coloration, sweetness, cell wall metabolism, and aroma that were coexpressed with these phytohormone signaling pathways (**Figure 4**). These included genes involved in sugar biosynthesis, such as *SPS*, *sucrose-6-phosphatase* (*SPP*) and *INV*, anthocyanin accumulation, HDMF formation, including *quinone oxidoreductase* (*QR*), which had a negative correlation with the modules indicating a positive relationship with receptacle ripening and were up-regulated by ABA, consistent with observed phenotypic changes (**Figures 1C**, **4**; **Supplementary Figures S1**, **S4**). In addition, Isoform0037314 and 0014205, which are positively related to turquoise module (**Supplementary Datasets S1**, **S2**) are homologs of FaSnRK2.6 and FaABI1, respectively, which are involved in ABA signaling and suppress receptacle ripening (Jia et al., 2013b; Han et al., 2015), and they were down-regulated by ABA (**Supplementary Figure S4**).

**Figure 5:**
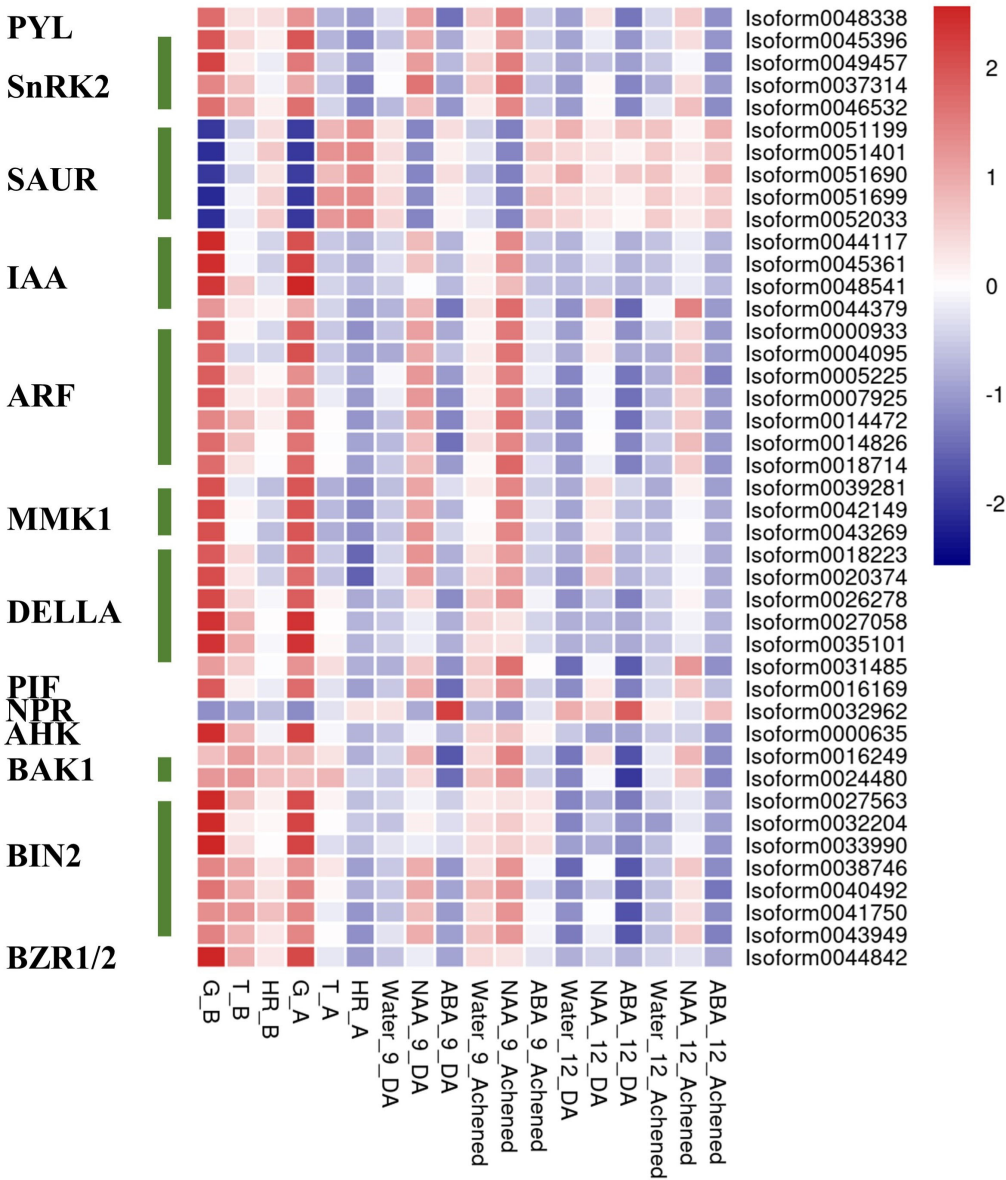
The expression profiles of hub phytohormone signaling genes in the receptacle during development and under treatments. The expression profiles of hub phytohormone signaling genes in the receptacle using heatmap analysis.

In summary, in this coexpression network, which consisted of five modules, were eight phytohormone signaling pathways. All GA signaling genes (25 isoforms), showed a negative association with receptacle ripening (**Figures 3**, **4**). Moreover, ABA, auxin, ethylene, JA, BR, and GA signaling pathways had at least 25 isoforms in the coexpression networks, which was considerably more than the corresponding numbers for cytokinin (CTK) and salicylic acid (SA) signaling (**Table 1**). Among these phytohormone signalings, 43 isoforms from seven signaling pathways, including ABA, auxin, ethylene, GA, BR, SA, and CTK, showed high correlation with their modules (correlation coefficient values > |± 0.9|), which suggested that they acted as hub phytohormone signalings to the most potentially participating in the receptacle ripening mediated by ABA (**Table 2**). In these hub signalings, the numbers of isoforms of Auxin and BR signaling pathways were at least ten while only several numbers were found in other pathways, including ABA. Notably, the most members of gene expression of hub phytohormone signalings were down-regulated by ABA, while only *small* auxin *up-regulated RNA* (*SAUR*) genes, belonged to auxin signaling pathway, and *a regulatory protein NPR* (*NPR*), belonged to SA signaling pathway, were up-regulated (**Figure 5**; **Table 2**). Among these hub phytohormone signalings, only FaSnRK2.6 (Isoform0037314) has been verified to be a negative regulator in receptacle ripening mediated by ABA, and the roles of others are still unclear.

**Table 2:** The list of isoforms of hub phytohormone signalings in receptacle ripening mediated by ABA.

Phytohormones	Gene name	ID	Modules	Correlation coefficient
ABA	PLY	Isoform0048338	turquoise	0.928147
	SnRK2	Isoform0045396	brown	-0.9327
		Isoform0049457	brown	-0.90388
		Isoform0037314	turquoise	0.913194
		Isoform0046532	turquoise	0.943011
Auxin	IAA	Isoform0044117	brown	-0.91165
		Isoform0045361	blue	0.901957
		Isoform0048541	blue	0.969533
		Isoform0044379	turquoise	0.909236
	ARF	Isoform0000933	brown	-0.93677
		Isoform0004095	brown	-0.92903
		Isoform0005225	turquoise	0.923927
		Isoform0007925	turquoise	0.914834
		Isoform0014472	turquoise	0.964573
		Isoform0014826	turquoise	0.944029
		Isoform0018714	turquoise	0.916653
	SAUR	Isoform0051199	brown	0.975982
		Isoform0051401	brown	0.954076
		Isoform0051690	brown	0.975727
		Isoform0051699	brown	0.951596
		Isoform0052033	brown	0.955492
Ethylene	MMK1	Isoform0039281	brown	-0.95045
		Isoform0042149	brown	-0.96244
		Isoform0043269	brown	-0.9588
GA	DELLA	Isoform0018223	brown	-0.91531
		Isoform0020374	brown	-0.91339
		Isoform0026278	blue	0.905991
		Isoform0027058	blue	0.964394
		Isoform0035101	blue	0.962586
		Isoform0031485	turquoise	0.908676
	PIF	Isoform0016169	turquoise	0.906372
SA	NPR	Isoform0032962	yellow	-0.90934
CTK	AHK	Isoform0000635	blue	0.93727
BR	BAK1	Isoform0016249	blue	0.908892
		Isoform0024480	yellow	0.900491
	BIN2	Isoform0027563	blue	0.908759
		Isoform0032204	blue	0.950279
		Isoform0033990	blue	0.916183
		Isoform0038746	turquoise	0.950838
		Isoform0040492	turquoise	0.94507
		Isoform0041750	turquoise	0.954566
		Isoform0043949	turquoise	0.975739
	BZR1/2	Isoform0044842	blue	0.933731

The abbreviations of gene names in the table as follow: PYL, abscisic acid receptor PYR/PYL family; SnRK2, subfamily 2 SNF1-related kinases; IAA, auxin-responsive protein IAA; ARF, auxin response factor; SAUR, small auxin up-regulated RNA; MMK1, MAPK KINASE1; DELLA, DELLA protein; PIF, phytochrome-interacting factor; NPR, regulatory protein NPR; AHK, Arabidopsis histidine kinase; BAK1, BRASSINOSTEROID INSENSITIVE 1-associated receptor kinase; BIN2, BRASSINOSTEROID INSENSITIVE 2; BZR1/2, BRASSINAZOLE RESISTANT 1/2. Red and blue in the correlation coefficient section represent expression profiles of isoforms positively and negatively related, respectively, to receptacle ripening mediated by ABA.

### Roles of FaSAURs, the hub phytohormone signalings, in receptacle ripening mediated by ABA

**Figure 6:**
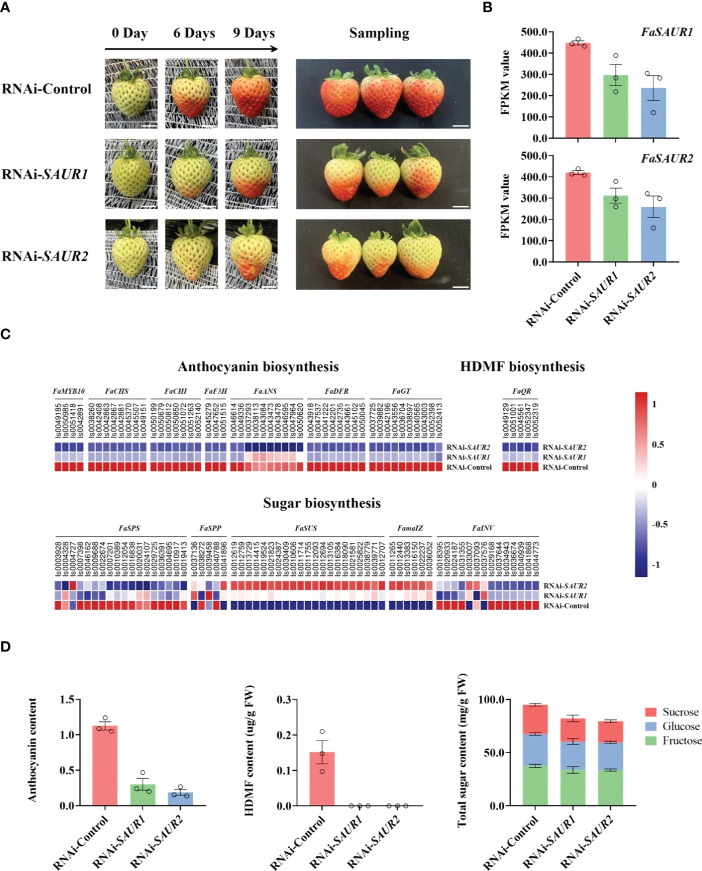
Transiently RNAi assays of *FaSAUR1* and *FaSAUR2* in strawberry fruit. **(A)** Phenotypes of fruit after transiently suppressing *FaSAUR1* or *FaSAUR2* using RNAi. The left picture shows the control (injecting empty vector, named as RNAi-Control) and RNAi (named RNAi-*FaSAUR1* and RNAi-*FaSARU2*) fruit at 0, 6, and 9 days, The right picture shows the fruit at sampling stage (9 days). Scale bar = 1 cm. **(B)** Expression levels of *FaSAUR1* or *FaSAUR2* in the control and RNAi fruit based on the FPKM of Isoform0051199 and 0051401. **(C)** Heatmap of expression profiles of genes involved in anthocyanin, HDMF, and sugar biosynthesis in the samples of transient assays, based on FPKM values. **(D)** Levels of anthocyanin, HDMF, and total sugars in the RNAi-Control and RNAi-*FaSAUR1* and RNAi-*FaSAUR2* fruit. The data represent the mean ± SD of three biological replicates.

Although Small auxin up-regulated RNA (SAUR) genes are important components of auxin signaling and participate in many aspects of plant growth and development (Ren & Gray, 2015), there is limited understanding of roles in non-climacteric fruit ripening. Among hub phytohormone signalings, five SAUR isoforms had the highest positively relationship with ripening-related quality traits, and were upregulated by ABA (**Figure 5**; **Table 2**), which suggested that they might act as a positive role in receptacle quality formation. To verify the prediction of the hub phytohormone singnaling network, we therefore firstly explored the function of these SAUR in receptacle ripening mediated by ABA. Based on an alignment of amino acid sequences, these isoforms were divided into FaSAUR1 (Isoform0051199 and 0051690) and FaSAUR2 (Isoform0051401, 0051699, and 0052033) sequences (**Supplementary Figure S5**). Difference among gene isoforms with the same amino acid sequence were observed in their untranslated regions (UTRs) (**Supplementary Figure S6**). Among these isoforms, the full-length mRNA sequences of Isoform0051199 and 0051401 were the longest in the *FaSAUR1* and *FaSAUR2* types, respectively (**Supplementary Figure S6**). To verify the function of *FaSAUR1* and *FaSAUR2*, RNAi targets, including the partial domains of CDS and 3’UTR, specific to each of the two genes were designed and used to silence each gene individually in strawberry fruit (**Supplementary Figure S5**). Transient RNAi assays showed that silencing *FaSAUR2* (RNAi-*FaSAUR2* fruit) generated a red area that was smaller than the areas caused by silencing *FaSARU1* (RNAi-*FaSAUR1* fruit) and both were less than in empty vector RNAi-Control fruit (**Figure 6A**). Notably, both *FaSAUR1* and *FaSAUR2* were silenced in RNAi-*FaSAUR1* and -*FaSAUR2* fruit, possibly due to sequence similarity between the two RNAi fragments (**Figure 6B**; **Supplementary Figures S5**, **S6**). Based on the transcriptome analysis, the expression levels of *FaSAUR1* and *FaSAUR2* in RNAi-*FaSAUR2* and RNAi-Control fruits were lowest and highest, respectively (**Figure 6B**). Moreover, the expressional levels of *FaMYB10* and the anthocyanin biosynthetic genes and the difference of anthocyanin content in the fruit corresponded with the fruit phenotypes and the expressional profiles of *FaSAUR1* and *FaSAUR2* (**Figure 6**). FaQR, a key gene in the biosynthesis of HDMF (Raab et al., 2006), was highly expressed in RNAi-Control fruit and was expressed at higher levels than in RNAi-*FaSAUR1* and *FaSAUR2* fruits, while HDMF was only was detected in RNAi-Control fruit (**Figure 6C, D**). In addition, most *FaSPS* genes, corresponding to the rate-limiting gene in sucrose biosynthesis, were down-regulated in the RNAi-*FaSAUR1* and -*FaSAUR2* fruits compared to RNAi-Control and their expressional levels in RNAi-*FaSAUR2* were higher than in RNAi-*FaSAUR1* (**Figure 6C**). Sucrose contents were approximately 23% and 31% lower in RNAi-*FaSAUR1* and *FaSAUR2* fruit, respectively, compared to RNAi-Control, which was likely the primary reason for the total sugar content in the control fruit being higher than that in RNAi-*FaSAUR1* and -*FaSAUR*2 fruits (**Figure 6C**).

## Discussion

ABA is widely described as a dominant regulator of non-climacteric fruit ripening (Fenn & Giovannoni, 2021). In strawberry fruit, ABA has also been found to interact with other phytohormones, including auxin, GAs, JAs, and ethylene, in receptacle ripening (Li et al., 2022a), suggesting multiple interlinked phytohormone signaling networks participate in ripening mediated by ABA. However, this idea has yet to be investigated in detail. Here we provide evidence of a coexpression network of phytohormone signals and hub signalings in the strawberry receptacle mediated by ABA, based on transcriptome profiling of the receptacle associated with changes in ABA levels, following removing achene and exogenous treatments, as well as data sets quantifying ripening compounds and associated phenotypes (**Supplementary Figure S2**).

The Previous study shows that the expression profiles of multiple genes of phytohormone signaling pathway, such as ABA, auxin, GA, and ethylene, are influenced in the receptacle with the changes of ABA levels (Gu et al., 2019), which suggests that ABA manipulates receptacle ripening not only *via* itself signaling pathway but also *via* controlling other phytohormone signalings. A total of 328 full-length mRNAs, including those associated with ABA, auxin, GA, JA, ethylene, BR, SA, and CTK signaling, TF genes, and genes related to coloration, sugar accumulation, softening, and aroma, were detected in this coexpression network (**Figures 3**, **4**). The clear coexpression relationship during receptacle ripening among these genes related to different phytohormone signaling components suggests them as regulators of this process. The expression profiles and sequences of these genes and others, totally 18,998, included in this coexpression network provide valuable datasets (**Supplementary Datasets S2**, **S3**) for studies of strawberry and potentially of other non-climacteric fruit. The coexpression network included homologs of *FaABI* (Jia et al., 2013) and *FaSnRK2.6* (Han et al., 2015), which negatively regulate receptacle ripening, and displayed decreasing expression levels with receptacle development and were down-regulated by ABA (**Figure 4A**; **Supplementary Figure S4**). Moreover, previous studies show that FaSHP (Daminato et al., 2013) and FaRIF (Martín-Pizarro et al., 2021) promote receptacle ripening and their expression is induced by ABA, while that of FaMADS1a (Lu et al., 2018) shows the opposite pattern. We identified homologs of these genes in our coexpression network and their predicted functions in receptacle ripening, based on the expressional profiles, were consistent with the previous studies (**Figures 3A**, **4B**; **Supplementary Figure S4**). Additionally, homologs of genes that have been shown to participate in strawberry fruit ripening and quality traits, such as *FaMYB10* (Kadomura-Ishikawa et al., 2015) and *FaQR* (Raab et al., 2006), were found in the coexpression network and their expressional profiles were consistent with changes in ripening phenotypes (**Figures 3**, **4**; **Supplementary Figure S4**; **Supplementary Dataset S1**). All of these results are consistent with high predictive power of the coexpression network, which provide many clues for studying mechanisms of strawberry receptacle ripening.

In addition, we identified 43 isoforms respectively belonged to seven phytohormone signaling pathways, including auxin, ABA, ethylene, GA, CTK, BR, and SA from the coexpression network. The expression of ABA (*PYL*, *SnRK2*s), auxin (*auxin response factor*s, *auxin-responsive protein IAA*s), ethylene (*MAPK KINASE1*s), GA (*DELLA*s, *phytochrome-interacting factor*), CTK (*arabidopsis histidine kinase*), and BR (*BRASSINOSTEROID INSENSITIVE 1-associated receptor kinase*s, BRASSINOSTEROID INSENSITIVE 2s, *BRASSINAZOLE RESISTANT 1/2*) signaling genes were negatively regulated by ABA (**Table 2**), which suggested that they might act as repressors for strawberry receptacle ripening. The most of them are not explored the function in the strawberry receptacle ripening but the homolog (Isoform0037314) of FaSnRK2.6. Additionally, FvMAPK3 (MITOGEN-ACTIVATED PROTEIN KINASE3) has been found to repress anthocyanin biosynthesis *via* phosphorylating CHALCONE SYNTHASE1 in wild strawberry *(F*. *vesca*) fruit (Mao et al., 2022). Although the homolog of FvMAPK3 was not found in the hub phytohormone signalings, three MMK1 belonging to MAPK cascades were identified and they also were predicted to negatively regulate ripening and quality formation. On the other hand, only auxin and SA pathways had the members, *SAUR*s and *NPR*, positively controlled by ABA, which suggested that they might promote quality formation in the receptacle. Based on these results, ABA promote receptacle ripening primarily through down- and up-regulating these hub phytohormone genes, which the specific roles in this process as an important point needs to be investigated in the future.

Using genes that emerged from the expression network analysis, we also extended knowledge of phytohormone signaling that modulates receptacle ripening mediated by ABA. Among the prediction of hub phytohormone signaling genes, only *SAUR*s and *NPR* were up-regulated by ABA, and the former showed the highest correlation coefficient with the ripening and ABA level (**Table 2**). Therefore, we firstly explored the function of SAURs in the receptacle ripening mediated by ABA, which also were used to verify the reliability of our prediction of hub signaling genes. SAURs are the largest family of genes that respond to auxin and their expression is also influenced by other phytohormones (Ren & Gray, 2015; Gu et al., 2019). However, the function of SAURs in non-climacteric ripening has yet been previously characterized. Recently, SlSAUR69 was found to promote tomato (*S. lycopersicum* cv. MicroTom) fruit ripening by altering the balance of auxin and ethylene (Shin et al., 2019). Based on predictions of the hub phytohormone signalings combining the analysis of amino acid sequence, expressional profiles and changes in receptacle quality traits, two SAUR homologs, FaSAUR1 and FaSAUR2, were identified as candidates of hub phytohormone signalings for participating in receptacle ripening mediated by ABA (**Figure 5**; **Supplementary Figures S5**, **S6**). We determined through transient RNAi assays and RNA-Seq that the anthocyanin, HDMF, and total sugar levels in RNAi-*FaSAUR1* and -*FaSAUR2* receptacles were lower than in RNAi-Control, in accordance with the changes in expression of the related genes (**Figure 6**). This is consistent with supposedly roles of *FaSAUR1* and *FaSAUR2* in positively regulating receptacle quality formation. Interestingly, a phylogenetic analysis indicated that FaSAUR1 and FaSAUR2 are closely related to AtSAUR76/77/78 (**Supplementary Figure S7**), which is a negative regulator of leaf growth (Markakis et al., 2013). Thus, these results further verified the predictive capacity of the coexpression network of multiple phytohormone signaling networks in receptacle ripening mediated by ABA. However, the specific mechanism of receptacle ripening mediated by FaSAUR1 and FaSAUR2 needs further study.

In summary, we describe a coexpression network of phytohormone signaling in the ripening receptacle mediated by ABA and present high-resolution expressional profiles and full-length RNA sequences of suites of genes included in this network (**Supplementary Datasets S2**, **S3**), and predict the hub phytohormone signaling genes involving in receptacle ripening mediated by ABA. In addition, we present a strategy for using these data to identify additional ripening factors from multiple phytohormone signaling systems and tested two auxin signaling pathway factors, *FaSAUR1* and *FaSAUR2*, which are up-regulated by ABA and that promote anthocyanin, HDMF, and sugar biosynthesis. These results have great potential for elucidating ripening and quality formation in strawberry receptacle with implications that can additionally be tested in other fruits.

## Data availability statement

The datasets presented in this study can be found in online repositories. The names of the repository/repositories and accession number(s) can be found below: https://ngdc.cncb.ac.cn/search/?dbId=gsa&amp;q=CRA006989&amp;page=1, CRA006989; https://ngdc.cncb.ac.cn/search/?dbId=gsa&amp;q=CRA006997&amp;page=1, CRA006997.

## Author contributions

K-SC and G-HJ managed the project. K-SC, JR, Y-NS and B-JL designed experiment and coordinated the project. K-SC, JR, Y-NS and B-JL wrote the paper. K-SC, JR, G-HJ, Y-NS, B-JL and JG discussed about the results of experiments and reviewed the paper. G-HJ and X-FY grew the plant material. B-JL, H-RJ, X-FY, Y-FS and JL collected and prepared samples. JL assisted B-JL in analyzing transcriptome data. H-RJ and Y-FS assisted B-JL in participating in the experiments. All authors contributed to the article and approved the submitted version.

## Supplementary material

Supplementary Data Sheet 1 The information of genes involved in phytohormone signalings, related to qualities, NAC and MADS families in five core modules.

Supplementary Data Sheet 2 All of full-length transcript sequences in five core modules.

Supplementary Data Sheet 3 The information of all of genes in five core modules.
