# Supplementary material for: Abscisic acid mediated strawberry receptacle ripening involves the interplay of multiple phytohormone signaling networks
Source: Front Plant Sci. 2023 Jan 30;14:1117156. doi: 10.3389/fpls.2023.1117156 (PMC9923025; doi:10.3389/fpls.2023.1117156)
Supplement: Supplementary file 2 [file DataSheet_2.docx]

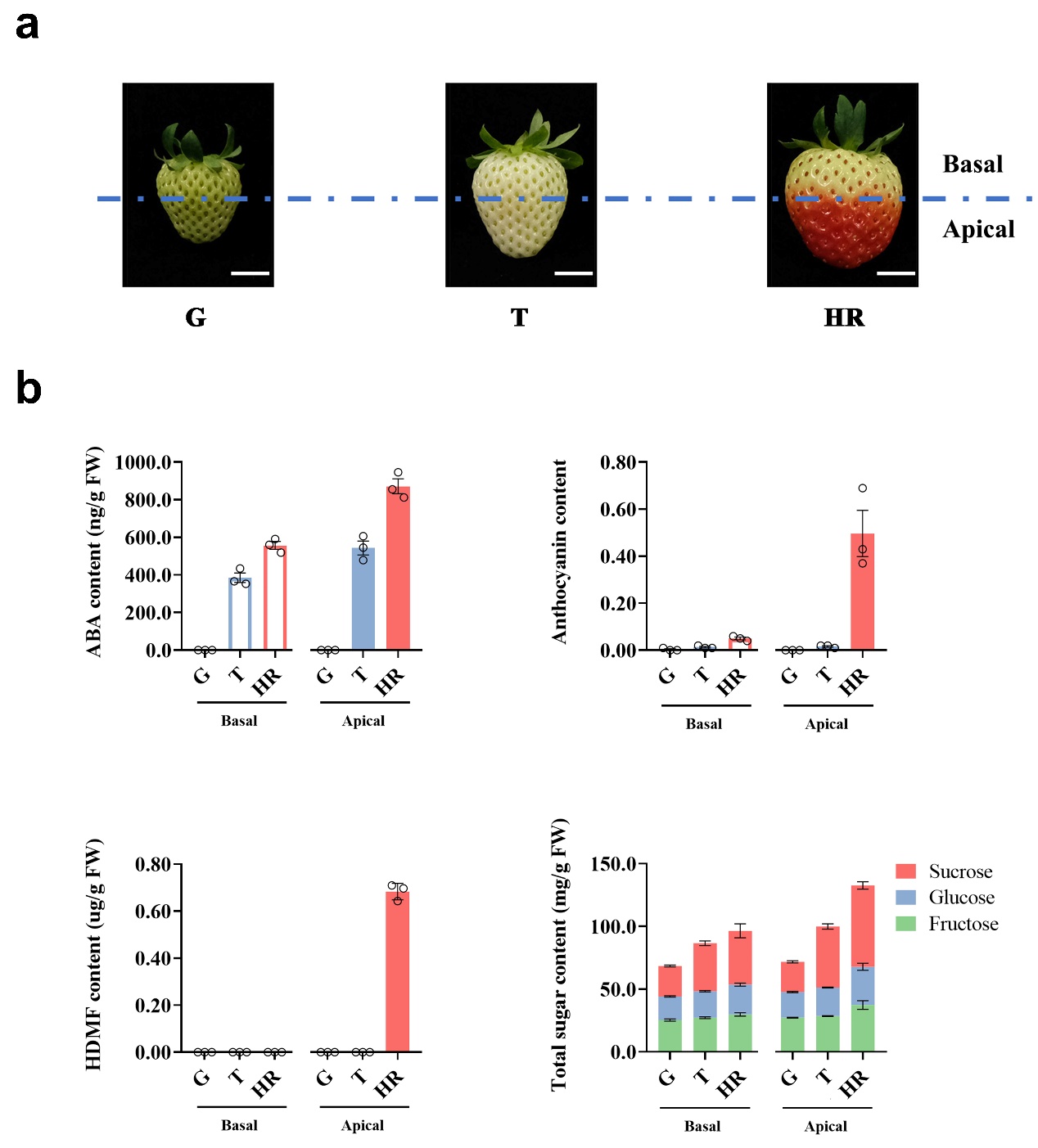


**Supplementary Figure S1** Levels of ABA and ripening-related compounds in different regions of the receptacle during development. **a** Phenotypes of strawberry fruit development, including green (G; fruit with green achenes and receptacle), turning (T; totally degreened achene and receptacle), and half-red stages (HR; half of the receptacle and all of achenes showing red coloration). Bar = 1 cm. **b** Measurements of ABA levels and fruit quality traits, including anthocyanin, 4-hydroxy-2,5-dimethyl-3(2)H-furanone (HDMF, i.e. furanone, a significant aroma compound in strawberry), and sugar, contents in the receptacle during development. The data are the mean ± SD from three biological replicates.


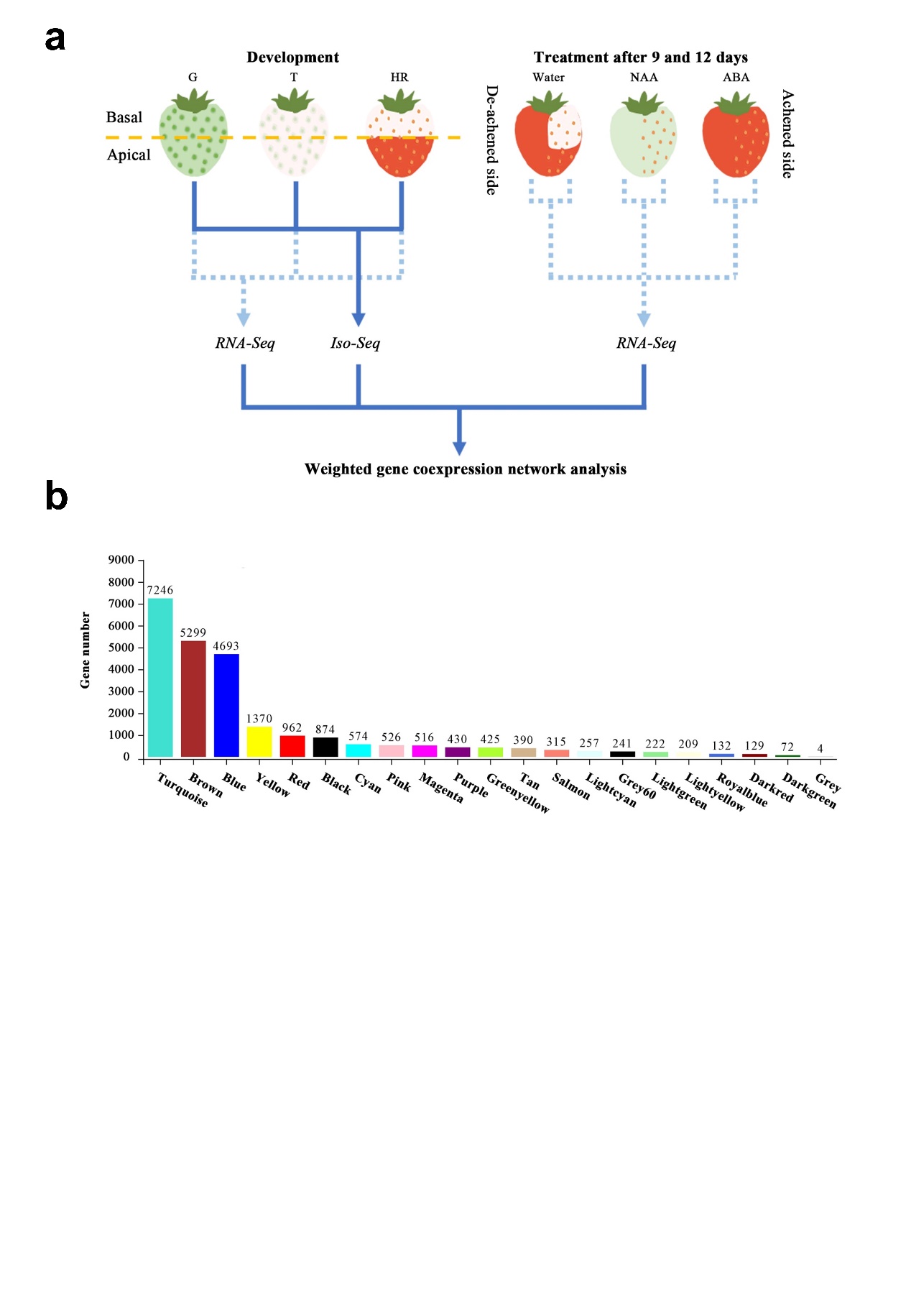


**Supplementary Figure S2** Strategy of transcriptome sequencing and construction of weighted gene coexpression network. **a** Experimental design and workflow for transcriptome sequencing and analysis. The mRNAs extracted from the basal and apical parts of the receptacle during development were pooled in equal amounts into one sample prior to Iso-Seq sequencing (PacBio) to generate the full-length transcripts (Isoforms). The different parts of the receptacle during development and under the treatments were used for RNA-Seq profiling (Illumina) to characterize the isoform expressional profiles. This information was used in the weighted gene coexpression network analysis (WGCNA). **b** The genes were clustered into 21 modules via WGCNA, and the numbers of isoforms in each module are indicated.


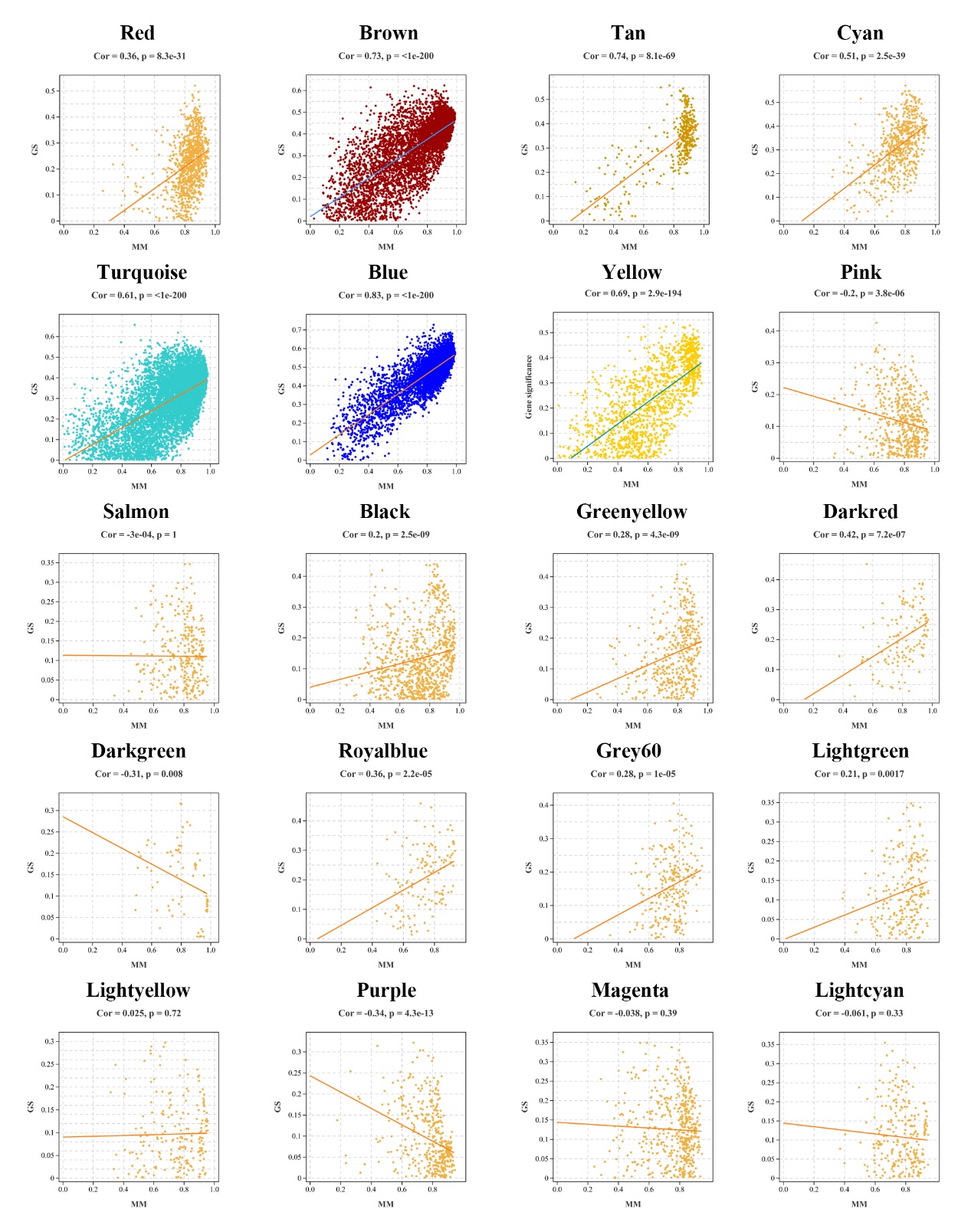


**Supplementary Figure S3** The analysis of correlation between gene significance, the correlation between each isoform and ABA level, and module membership, the correlation between expressional profile of each isoform and module. GS, gene significance. MM, module membership. *P* value were analyzed using Student’s paired *t*-test.


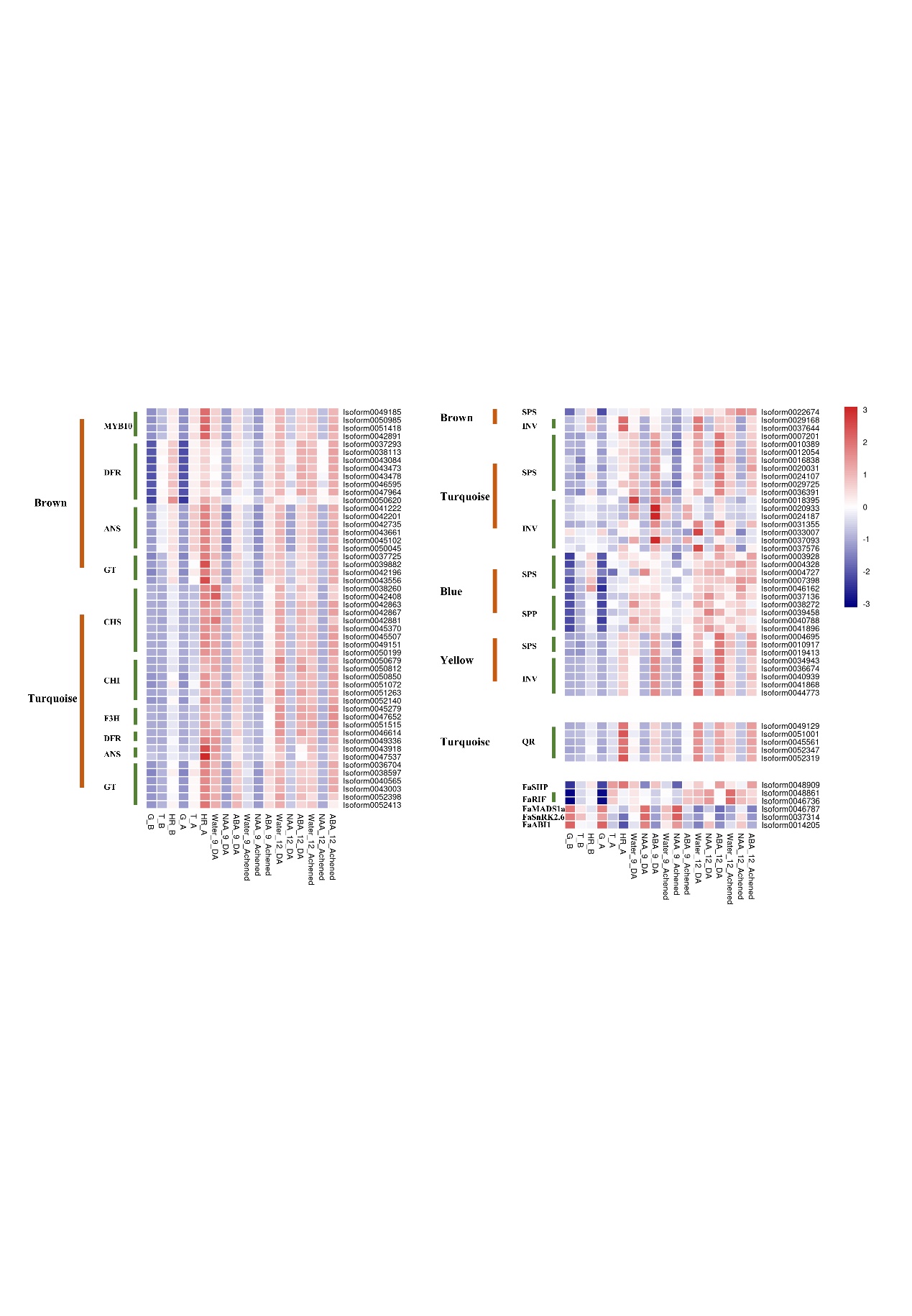


**Supplementary Figure S4** The expressional profiles of genes related to ripening and quality formation in receptacle during development and under the treatments based on their FPKM. The data are the mean from three biological replicates. The abbreviations of gene names in the table as follow. Anthocyanin biosynthesis pathway: DFR, dihydroflavonol 4-reductase; ANS, anthocyanidin synthase; GT, anthocyanidin 3-*O*-glucosyltransferase; CHS, chalcone synthase; CHI, chalcone isomerase; F3H, flavanone 3-hydroxylase. Sugar biosynthesis pathway: SPS, sucrose-phosphate synthase; INV, beta-fructofuranosidase/invertase; SPP, sucrose-6-phosphatase. HDMF biosynthesis pathway: QR, quinone oxidoreductase.


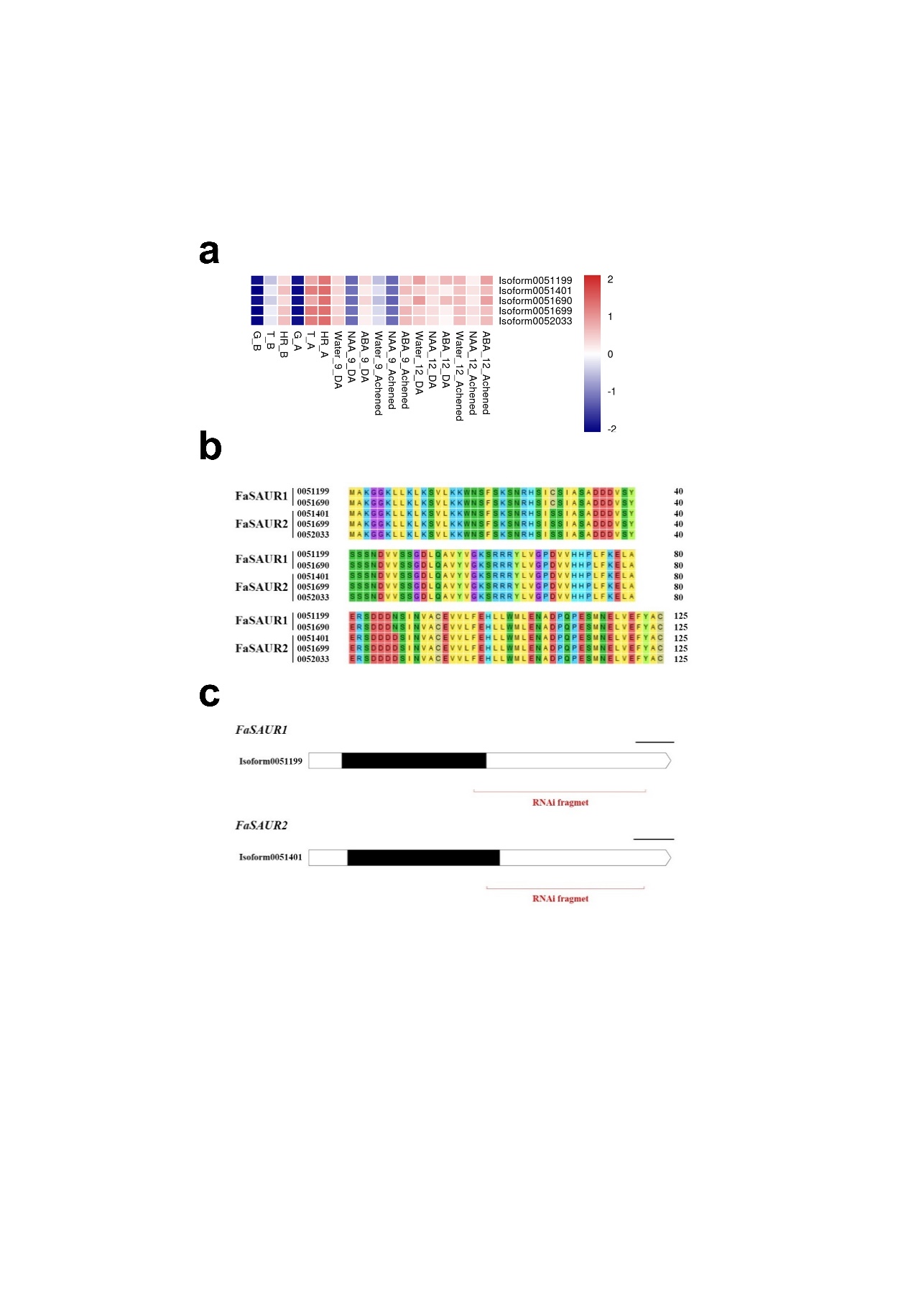


**Supplementary Figure S5** Characterization of expressional profiles and sequences of *FaSAUR1* and *FaSAUR2*. **a** The expression profiles of isoforms annotated as SAUR genes. These isoforms had a similar expressional profile. **b** The alignment of amino acid sequences of isoforms. Based on the discrepancy of the amino acid sequences, these isoforms were divided into two types: FaSAUR1 and FaSAUR2. **c** The full-length transcript sequences of Isoform005119 and 0051401 were the longest in *FaSAUR1* and *FaSAUR2*, respectively. RNAi (RNA inhibition) fragments of *FaSAUR1* and *FaSAUR2* were designed were based on these sequences. The red bracket represents the RNAi fragment in *FaSAUR*s.


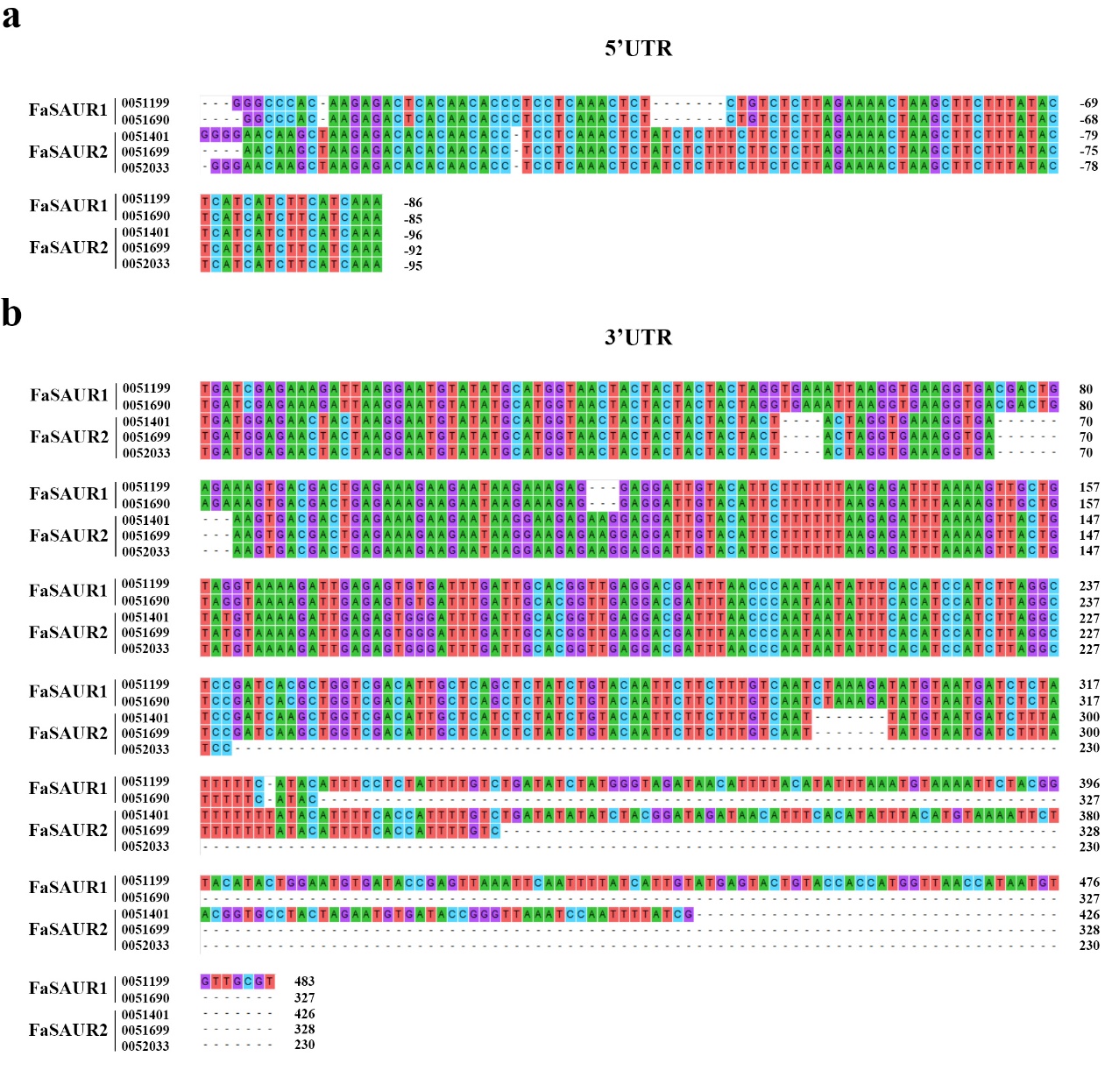


**Supplementary Figure S6** The alignments of UTR sequences among *FaSAUR*s. **a** The alignment of 5’UTR sequences among *FaSAUR*s. **b** The alignment of 3’UTR sequences among *FaSAUR*s.


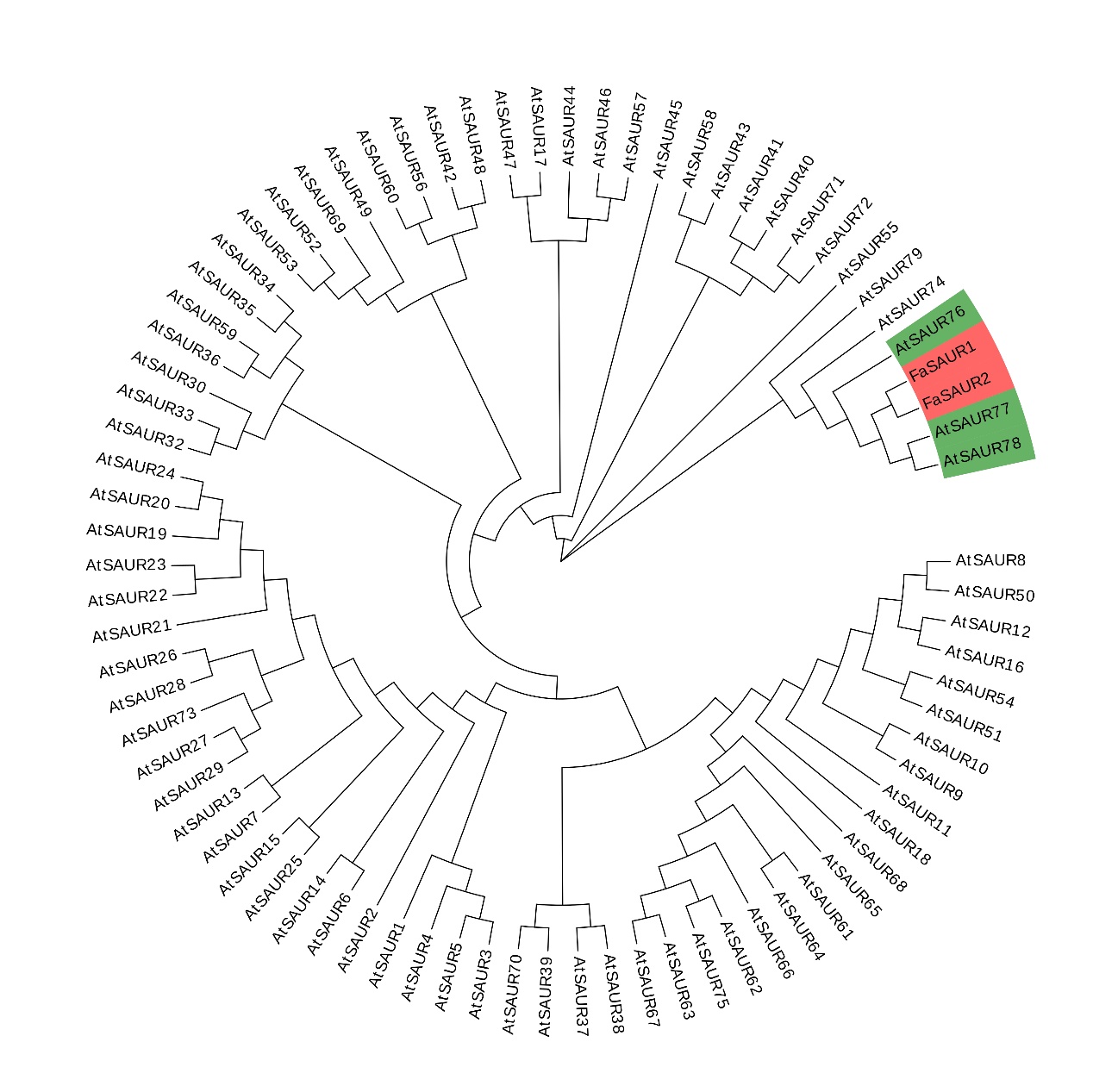


**Supplementary Figure S7** All amino acid sequences of AtSAURs from *Arabidopsis thaliana* and FaSAUR1/2 were used to build a phylogenetic tree based on the neighbour-joining method. FaSAUR1/2 with red colour in the picture were closest to AtSAUR76/77/78 marked green colour based on the NJ phylogenetic tree. All of amino acid sequences of AtSAURs were downloaded from [www.arabidopsis.org](http://www.arabidopsis.org), and their gene ID as follow: AtSAUR1 (AT4G34770), AtSAUR2 (AT4G34780), AtSAUR3 (AT4G34790), AtSAUR4 (AT4G34800), AtSAUR5 (AT4G34810), AtSAUR6 (AT2G21210), AtSAUR7 (AT2G21200), AtSAUR8 (AT2G16580.1), AtSAUR9 (AT4G36110), AtSAUR10 (AT2G18010.1), AtSAUR11 (AT5G66260), AtSAUR12 (AT2G21220), AtSAUR13 (AT4G38825), AtSAUR14 (AT4G38840), AtSAUR15 (AT4G38850), AtSAUR16 (AT4G38860), AtSAUR17 (AT4G09530), AtSAUR18 (AT3G51200), AtSAUR19 (AT5G18010), AtSAUR20 (AT5G18020), AtSAUR21 (AT5G18030), AtSAUR22 (AT5G18050), AtSAUR23 (AT5G18060), AtSAUR24 (AT5G18080), AtSAUR25 (AT4G13790), AtSAUR26 (AT3G03850), AtSAUR27 (AT3G03840), AtSAUR28 (AT3G03830), AtSARU29 (AT3G03820), AtSAUR30 (AT5G53590), AtSAUR32 (AT2G46690), AtSAUR33 (AT3G61900), AtSAUR34 (AT4G22620), AtSAUR35 (AT4G12410), AtSAUR36 (AT2G45210), AtSAUR37 (AT4G31320), AtSAUR38 (AT2G24400), AtSAUR39 (AT3G43120), AtSAUR40 (AT1G79130.1), AtSAUR41 (AT1G16510.1), AtSAUR42 (AT2G28085), AtSAUR43 (AT5G42410), AtSAUR44 (AT5G03310), AtSAUR45 (AT2G36210), AtSAUR46 (AT2G37030), AtSAUR47 (AT3G20220), AtSAUR48 (AT3G09870), AtSAUR49 (AT4G34750), AtSAUR50 (AT4G34760), AtSAUR51 (AT1G75580.1), AtSAUR52 (AT1G75590.1), AtSAUR53 (AT1G19840.1), AtSAUR54 (AT1G19830.1), AtSAUR55 (AT5G50760), AtSAUR56 (AT1G76190.1), AtSAUR57 (AT3G53250), AtSAUR58 (AT1G43040.1), AtSAUR59 (AT3G60690), AtSAUR60 (AT1G20470.1), AtSAUR61 (AT1G29420.1), AtSAUR62 (AT1G29430.1), AtSAUR63 (AT1G29440.1), AtSAUR64 (AT1G29450.1), AtSAUR65 (AT1G29460.1), AtSAUR66 (AT1G29500.1), AtSAUR67 (AT1G29510.1), AtSAUR68 (AT1G29490.1), AtSAUR69 (AT5G10990), AtSAUR70 (AT5G20810), AtSAUR71 (AT1G56150.1), AtSAUR72 (AT3G12830), AtSAUR73 (AT3G03847), AtSAUR74 (AT3G12955), AtSAUR75 (AT5G27780), AtSAUR76 (AT5G20820), AtSAUR77 (AT1G17345.1), AtSAUR78 (AT1G72430.1), AtSAUR79 (AT2G35290).
