# Supplementary material for: Abscisic acid mediated strawberry receptacle ripening involves the interplay of multiple phytohormone signaling networks
Source: Front Plant Sci. 2023 Jan 30;14:1117156. doi: 10.3389/fpls.2023.1117156 (PMC9923025; doi:10.3389/fpls.2023.1117156)
Supplement: Supplementary file 3 [file Table_1.docx]

**Supplementar**y **Table 1** Primers of cloning RNAi fragments used in this study.

| Primer name | Sequence (5’-3’) |
| --- | --- |
| FaSAUR1_RNAi_F | GGGGACAAGTTTGTACAAAAAAGCAGGCTGAACGAGCTCGTCGAGTT |
| FaSAUR1_RNAi_R | GGGGACCACTTTGTACAAGAAAGCTGGGTCGGTATCACATTCCAGTATGTACCG |
| FaSAUR2_RNAi_F | GGGGACAAGTTTGTACAAAAAAGCAGGCTGAACGAGCTCGTCGAGTTCT |
| FaSAUR2_RNAi_R | GGGGACCACTTTGTACAAGAAAGCTGGGTGTGAAATGTTATCTATCCGTAGATA |
